# Prdm1 (Blimp-1) and the Expression of Fast and Slow Myosin Heavy Chain Isoforms during Avian Myogenesis *In Vitro*

**DOI:** 10.1371/journal.pone.0009951

**Published:** 2010-04-01

**Authors:** Mary Lou Beermann, Magdalena Ardelt, Mahasweta Girgenrath, Jeffrey Boone Miller

**Affiliations:** 1 Neuromuscular Biology & Disease Group, Boston Biomedical Research Institute, Watertown, Massachusetts, United States of America; 2 Senator Paul D. Wellstone Muscular Dystrophy Cooperative Research Center, Boston Biomedical Research Institute, Watertown, Massachusetts, United States of America; 3 Department of Health Science, Boston University, Boston, Massachusetts, United States of America; 4 Department of Neurology, Harvard Medical School, Boston, Massachusetts, United States of America; Texas A&M University, United States of America

## Abstract

**Background:**

Multiple types of fast and slow skeletal muscle fibers form during early embryogenesis in vertebrates. In zebrafish, formation of the earliest slow myofibers in fin muscles requires expression of the zinc-finger transcriptional repressor Prdm1 (also known as Blimp1). To further understand how the role of Prdm1 in early myogenesis may vary through evolution and during development, we have now analyzed Prdm1 expression in the diverse types of myotubes that form in culture from somitic, embryonic, and fetal chicken myoblasts.

**Principal Findings:**

In cultures of somitic, embryonic limb, and fetal limb chicken cells, we found that Prdm1 was expressed in all of the differentiated muscle cells that formed, including those that expressed only fast myosin heavy chain isoforms, as well as those that co-expressed both fast and slow myosin heavy chain isoforms. Prdm1 was also expressed in Pax7-positive myoblasts, as well as in non-myogenic cells in the cultures. Furthermore, though all differentiated cells in control somite cultures co-expressed fast and slow myosin heavy chains, antisense knockdown of Prdm1 expression inhibited the formation of these co-expressing cells in somite cultures.

**Conclusions:**

In chicken myogenic cell cultures, Prdm1 was expressed in most Pax7-positive myoblasts and in all differentiated muscle cells, irrespective of the developmental stage of cell donor or the pattern of fast and slow myosin heavy chains expressed in the differentiated cells that were formed. Thus, Prdm1 was expressed in myogenic cells prior to terminal differentiation; and, after differentiation, Prdm1 expression was not limited to cells that expressed slow myosin heavy chain isoforms. In addition, Prdm1 appeared to be required for differentiation of the somitic myocytes, which are the earliest myocytes to form in the avian embryo.

## Introduction

In developing vertebrates, distinct types of fast and slow myofibers form during embryonic and fetal development. One marker for this myofiber diversity is differential expression of fast and slow isoforms of myosin heavy chain (MyHC). Recent work with several animal models has begun to uncover molecular and cellular mechanisms that regulate the formation of distinct types of fast and slow myofibers. As one example, studies with zebrafish mutants have shown that the zinc finger protein Prdm1 (also known as Blimp1) is required for formation of the first population of slow MyHC-expressing myocytes that form during development [1], [2]. The expression pattern of Prdm1 in lamprey somites is compatible with a similar function [3]. In the mouse, Prdm1 is known to function in the differentiation of multiple non-myogenic cell lineages and is expressed in somites, though analyses of slow muscle formation have not been reported in Prdm1-null mice [4]–[6]. Because it was not known if Prdm1 was required for slow muscle formation in vertebrates other than teleost fish, we have now examined Prdm1 expression and function in differentiating skeletal muscle cells from the chicken.

As in chickens and other vertebrates, zebrafish myogenesis proceeds through multiple cellular stages to produce the final complement of skeletal muscles [7]. The first slow myofibers in the zebrafish form from adaxial cells of the somites in response to hedgehog (Hh) signaling, and, in these cells, Prdm1 appears to promote the slow phenotype both by directly repressing fast muscle genes and by lifting Sox6-mediated repression of slow muscle genes [8]–[10]. Prdm1 also is required for formation of an additional group of superficial slow myofibers, though this process is independent of Hh signaling, and, furthermore, many slow fibers form in the zebrafish independently of Prdm1 [7]. The hedeghog (Hh) family proteins, particularly sonic hedgehog (Shh), regulate expression of the Gli family of zinc finger transcription factors [11]–[14] that in turn regulate expression of the muscle regulatory factors (MRFs) including Myf5 and MyoD [15].

Myogenesis in the developing chicken embryo proceeds through distinct stages in which multiple types of myoblasts and myofibers appear [16]–[20]. The first differentiated muscle cells appear in the myotomal compartment of the rostral somites by Hamburger-Hamilton (HH) stage 14 on embryonic day 2 (E2); and these somitic myocytes begin to co-express both fast and slow isoforms of MyHC shortly after they form [21]. In chicken embryo limb buds, the first myofibers begin to form by E3 – E4, and these primary myofibers are of at least two distinct types: a fast type that expresses only fast MyHC(s) and a fast/slow type that co-expresses both fast and slow MyHCs [22]. This initial diversification of fast and fast/slow myofibers does not depend on functional innervation [18], [22], [23]. Embryonic chicken limbs also contain distinct types of myoblasts that are committed to form either fast or fast/slow myotubes [24]–[27]. As fetal development begins on ∼E8, a distinct population of fetal myoblasts appears and secondary myofibers are formed alongside the primary fibers in the limbs [23], [28].

To begin to determine the possible role of Prdm1 in avian myogenesis, we have now analyzed the pattern of Prdm1 expression in cultures of myogenic cells obtained from the somites, embryonic limbs, and fetal limbs of developing chickens. We found that Prdm1 was expressed in all of these different types of myogenic cells and was not limited to differentiated muscle cells that expressed slow MyHC. In addition, we found that antisense knockdown of Prdm1 inhibited MyHC expression in somite cultures, suggesting that Prdm1 has a functional role in chicken myogenesis.

## Results

**Figure 1:**
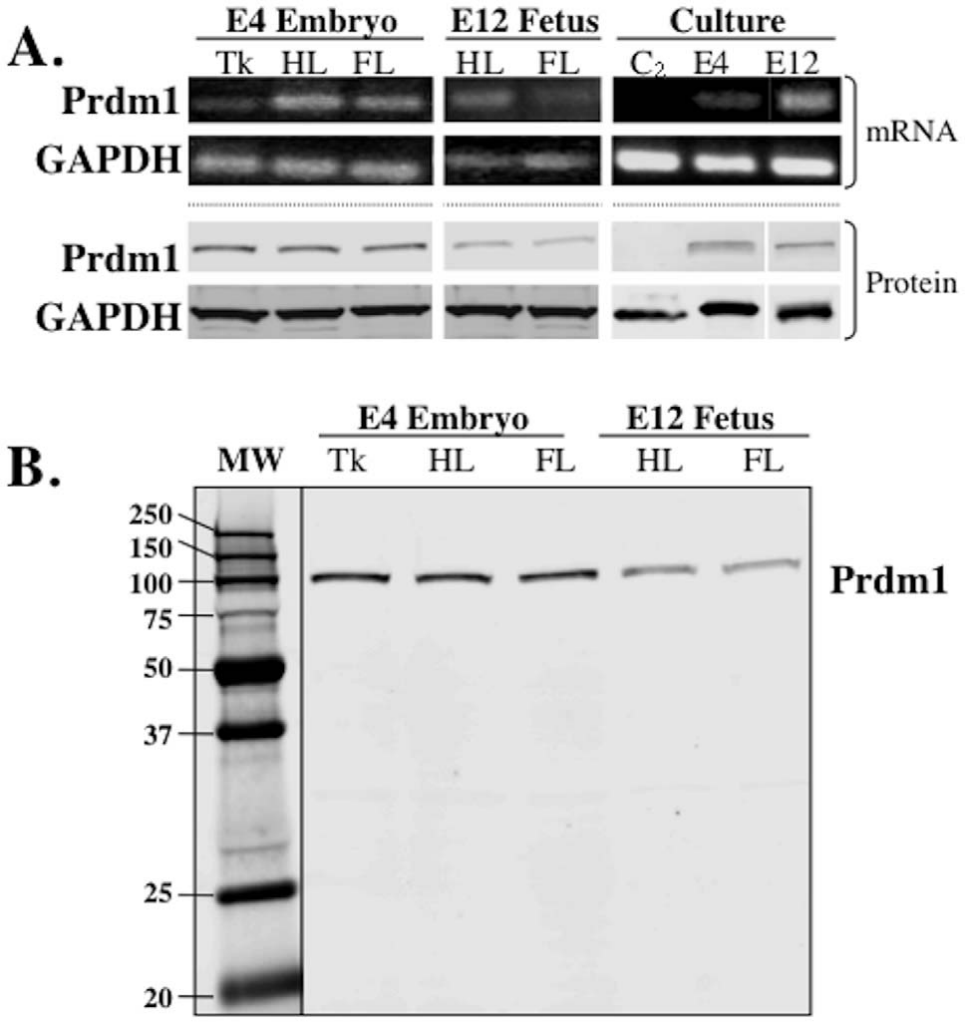
Prdm1 mRNA and protein were expressed in chicken tissues and muscle cell cultures. **A.** As indicated, RNAs or proteins were isolated from trunk tissues (Tk), hindlimbs (HL), and forelimbs (FL) of chicken embryos (E4  =  day 4 *in ovo*) and fetuses (E12), and also from differentiated cultures of mouse C_2_C_12_ myogenic cells (C_2_) and primary cultures of E4 and E12 chicken hindlimb cells. RNAs were analyzed by RT-PCR with exon-spanning primers specific for chicken Prdm1 and, as a positive control, GAPDH as indicated. Proteins were analyzed by immunoblotting with antibodies specific for Prdm1 (Cell Signaling Technology) or GAPDH. Bands with the expected ∼240 bp size of the Prdm1 RT-PCR product or the expected ∼100 kDa M_r_ of the Prdm1 protein were obtained from all chicken embryonic and fetal tissues and from both E4 and E12 chicken myogenic cell cultures. Prdm1 mRNA and protein were not obtained from cells of the mouse C_2_C_12_ myogenic cell line, though GAPDH was found. Specificity of the amplified cDNAs for Prdm1 was confirmed by restriction enzyme analysis (not shown). Bands from some gels were re-arranged for presentation. **B.** Full-length Prdm1 immunoblot (Ab from Cell Signaling Technology) of the same tissue samples shown in panel A, demonstrating lack of non-specific bands. MW  =  molecular standards with number  =  kDa.

Using RT-PCR and immunoblotting, we first found that Prdm1 mRNA and protein was detectable at E4 and E12 in each of the different regions of the embryo that we assayed (Fig. 1). By RT-PCR, we detected Prdm1 mRNA in E4 trunk, hindlimb, and forelimb, and E12 hindlimb and forelimb tissues (Fig. 1A, top). The PCR primers spanned a splicing site in the Prdm1 mRNA and were designed to produce a 240 bp cDNA. Restriction analysis of the amplified cDNA was used to confirm identity of the amplification product (not shown). The PCR primers, which were specific for chicken Prdm1, did not amplify a product from cultures of the mouse C_2_C_12_ myogenic cell line, but Prdm1 mRNA was found in cultures of chicken E4 and E12 hindlimb cells (Fig. 1A, top). By immunoblot, we detected an immunoreactive band with M_r_ of ∼100 kDa as expected for the Prdm1 protein in the same chicken tissues and cell cultures in which we detected Prdm1 mRNA (Figs. 1A and 1B). Both the Cell Signal Technology and Abcam antibodies detected the ∼100 kDa band. Though the anti-Prdm1 antibody (Abcam) did react with Prdm1 in a sample of mouse thymus tissue (not shown), no Prdm1 protein was detectable in mouse C_2_C_12_ cell cultures.

Having detected Prdm1 at E4 and E12 in regions of the embryo in which myogenesis occurs, we next used immunocytochemistry to determine the expression pattern of Prdm1 in cultures of cells obtained from these different regions and developmental stages. Specifically, to study Prdm1 expression in the different types of somitic, embryonic, and fetal myoblasts, we examined cultures of cells prepared from E4 somites, E4 limbs, and E12 limbs (see Methods). As described below, we found Prdm1 to be expressed in each of the different types of somitic, embryonic limb, and fetal limb myogenic cells.

**Figure 2:**
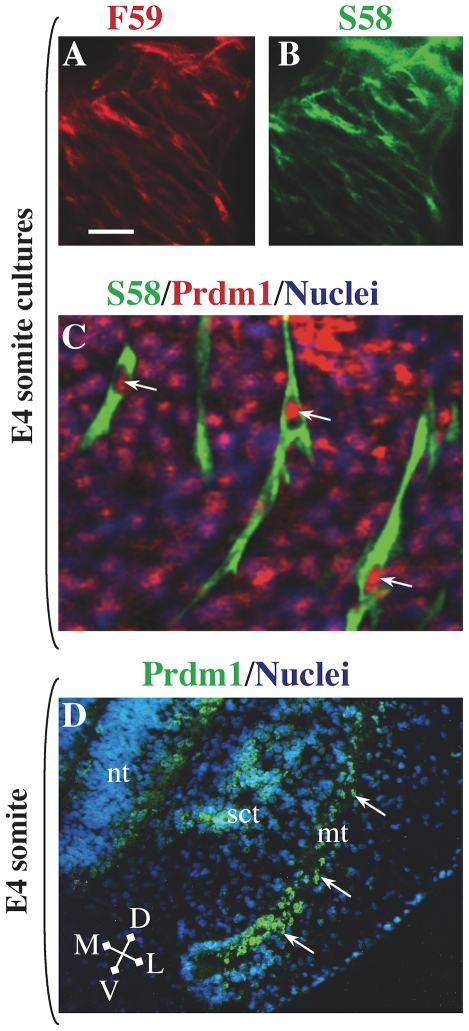
Expression of Prdm1 and fast and slow MyHC isoforms in E4 somite explant cultures and the somitic myotome. Double immunofluorescence analysis for fast MyHC(s) with mAb F59 (**A**, red fluorescence) and slow MyHC(s) with mAb S58 (**B**, green fluorescence) showed that fast and slow MyHCs were co-expressed by all differentiated myocytes in somite cultures. In addition, Prdm1 immunostaining (Ab from Cell Signaling Technology) was found in the nuclei of all myocytes, as well as in many MyHC-negative cells (**C**, merged image, green fluorescence  =  S58, red fluorescence  =  Prdm1, blue fluorescence  =  nuclei). **D**. Prdm1 (green fluorescence, Ab from Abcam) was expressed throughout the myotome (mt, arrows) of a mature somite at the forelimb bud level at E4. Additional Prdm1 staining was found in some cells of the sclerotome (sct) and neural tube (nt). Arrows indicate Prdm1-positive nuclei within myocytes. Bar in Panel A = 40 µm for panels A and B); 15 µm for panel C; and 50 µm for panel D.

In cultures of somite-derived cells, we found that the MyHC-expressing cells were mononucleate and that all of these myocytes co-expressed fast MyHC(s), slow MyHC(s), and Prdm1 (Fig. 2). In particular, after 1–2 days of differentiation, all somite-derived myocytes immunostained with both mAb F59, which reacts with all fast class MyHC isoforms (embryonic, neonatal, and all adult isoforms) and mAb S58, which reacts strongly with the slow MyHC2 and slow MyHC3 isoforms and weakly with the slow MyHC1 isoform (Fig. 2A, B) [21], [29]. We also found that Prdm1 was expressed in the nuclei of all somitic myocytes (Fig. 2C). We found this same pattern of Prdm1 nuclear immunostaining with three different antibodies that were prepared against two different epitopes that are conserved between human and chicken Prdm1 (see Methods for details of antibodies). Prdm1 expression was not limited to differentiated myocytes in the somite cultures, as most MyHC-negative cells in these cultures also showed nuclear staining for Prdm1 (Fig. 2C).

**Figure 3:**
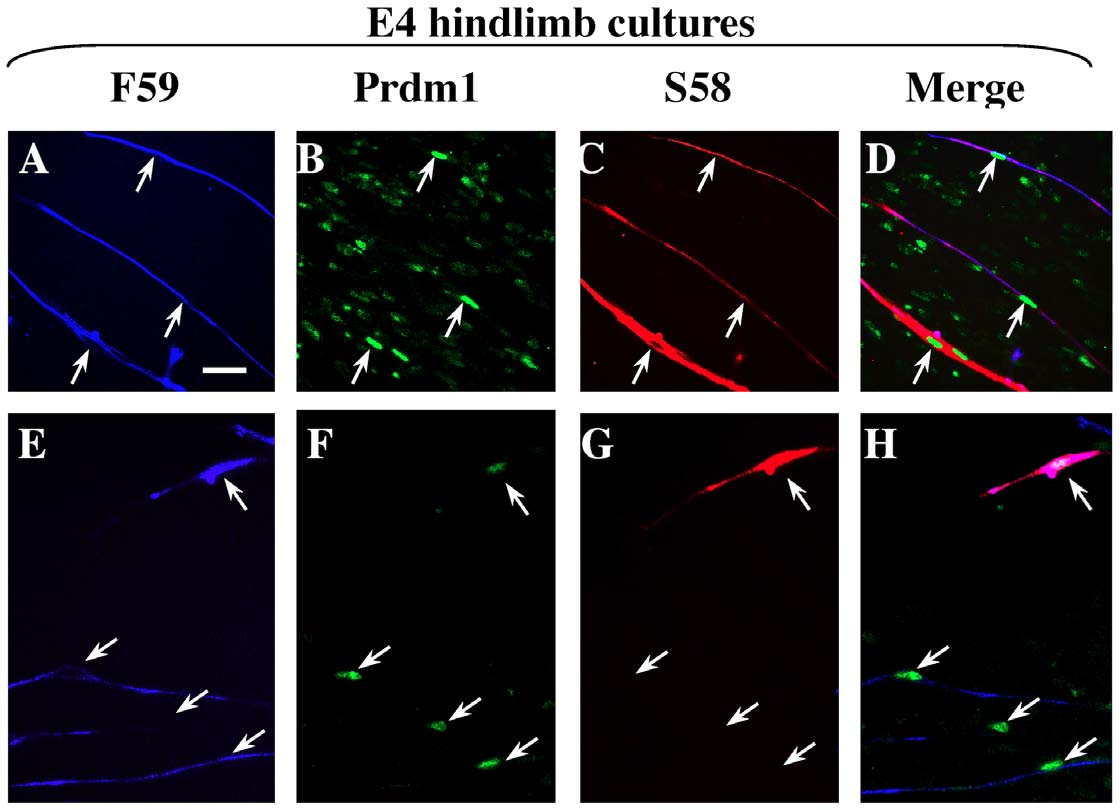
Expression of Prdm1 and fast and slow MyHC isoforms in cultures of E4 hindlimb cultures. As indicated, individual cultures were analyzed by triple immunostaining for fast MyHC(s) with mAb F59 (A, E, blue fluorescence), Prdm1 (B, F, green fluorescence, Ab from Cell Signaling Technology), and slow MyHC(s) with mAb S58 (C, G, red fluorescence). Merged photographs (D, H) showed that, as found previously, E4 myoblasts formed differentiated, usually mononucleate, cells of two types: those that express only fast MyHC(s) (*e.g.*, downward pointing arrows in panels E–H) and those that co-express fast and slow MyHCs (upward pointing arrows in all panels). Prdm1 immunostaining was found in the nuclei of both types of differentiated cells. An additional Prdm1 antibody (Abcam) produced similar nuclear staining of E4 cultures (not shown). Bar in Panel A = 20 µm.

We also found that Prdm1 was expressed in the different fast and fast/slow types of MyHC-expressing cells that formed in cultures of E4 hindlimb and forelimb bud cells. As found previously [16], [30], the differentiated muscle cells formed in E4 limb bud cultures after 2–4 days of differentiation were small (1–3 nuclei). Also consistent with previous studies [24], [25], [30], about two-thirds of the differentiated cells were of the fast type that reacted with mAb F59 but not mAb S58 and thus expressed only fast MyHC(s), whereas the remaining differentiated cells were of the fast/slow type that reacted with both mAbs F59 and S58 and thus co-expressed both fast and slow MyHCs (Fig. 3). A previous study had shown that the fast/slow type of differentiated cells also express the slow MyHC1 isoform in addition to the S58-reactive slow MyHC2/3, but that none of the three slow MyHCs is expressed in the fast type of embryonic myotubes in culture [29]. By immunostaining, we found that Prdm1 was expressed in the nuclei of both the fast and fast/slow types of differentiated cells in the E4 limb cultures (Fig. 3). Prdm1 was also expressed in most of the cells that did not express MyHC (*e. g.*, Fig. 3B). These results, which were obtained with both the Cell Signal Technology and Abcam antibodies, showed that Prdm1 expression in differentiated E4 limb cells was found in cells that expressed only fast MyHC(s), as well as in those that co-expressed fast and slow MyHC(s).

**Figure 4:**
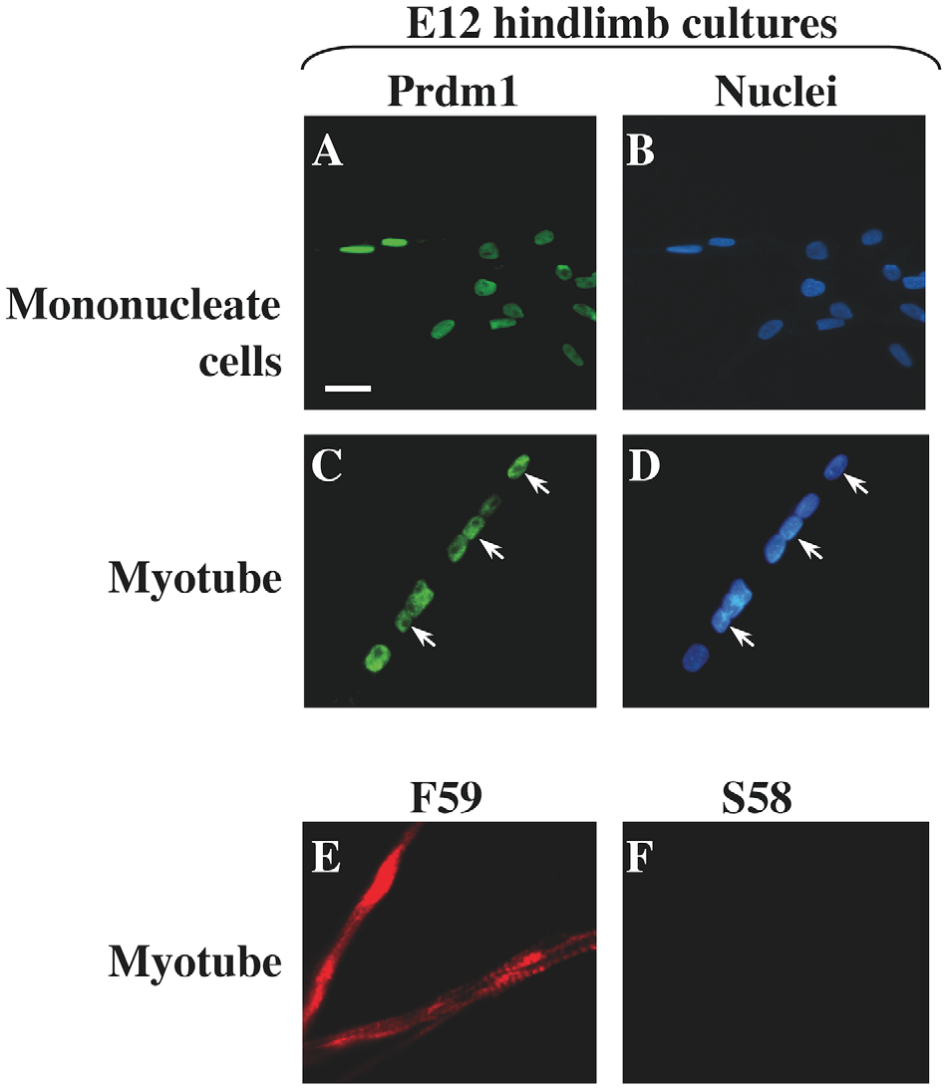
Prdm1 and fast MyHC expression in cultures of E12 hindlimb cells. Prdm1 immunostaining (Ab from Cell Signaling Technology) was found in the nuclei of both mononucleate cells (**A, B**) and myotubes (**C, D**). An additional Prdm1 antibody (Abcam) produced similar nuclear staining of E12 mononucleate cells and myotubes (not shown). As found in previous studies, myotubes formed from E12 myoblasts showed immunostaining with mAb F59 (**E**), which reacts with all fast MyHC isoforms, but did not stain with mAb S58 (**F**), which reacts with the slow MyHC2 and slow MyHC3 isoforms. Bar in Panel A = 10 µm for panels A–D and 30 µm for panels E and F.

We also found that Prdm1 was expressed in the myotubes formed from fetal E12 limb myoblasts (Fig. 4). As in previous work [16], [30], we found that fetal E12 myoblasts formed very large, multinucleate myotubes that reacted with mAb F59 but did not react with mAb S58 after 3–4 days in differentiation medium (Fig. 4E, F). Furthermore, previous work had shown that the slow MyHC1 isoform is not expressed by myotubes in such E12 cultures until at least 7–10 days after myotube formation and then only in a subset of the myotubes [30], [31]. Thus, when we analyzed fetal cultures after 3–4 days of differentiation, these fetal myotubes expressed only fast MyHC(s). We found that Prdm1 immunostaining in all nuclei within the multinucleate fetal myotubes (Fig. 4C, D), as well as in mononucleate cells in the cultures (Fig. 4A, B). Both the Cell Signal Technology and Abcam antibodies gave this result. Thus, as in the embryonic E4 cultures, Prdm1 was expressed in differentiated cells that expressed only fast MyHC(s).

**Figure 5:**
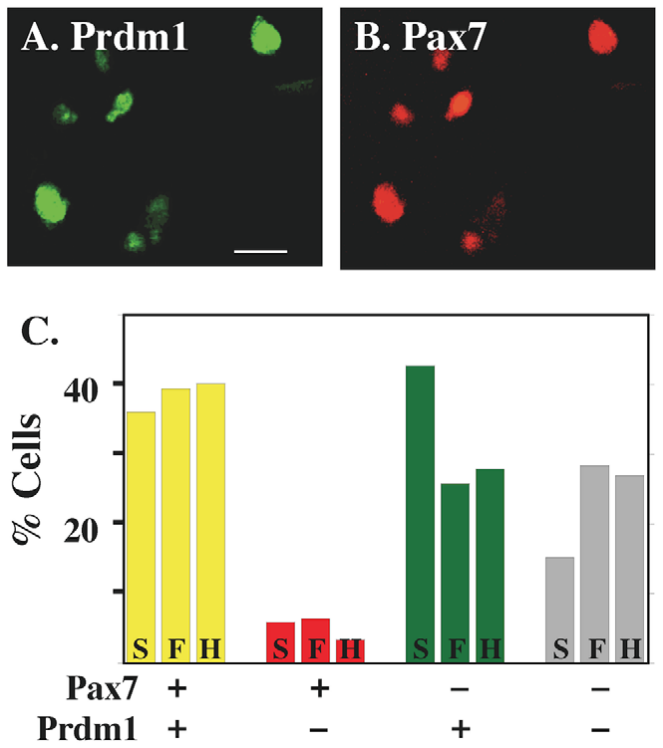
Expression of Prdm1 and Pax7 in E4 somite and limb cell cultures. As indicated, individual cultures, in this case a proliferating E4 hindlimb culture prior to myotube formation, were analyzed by double immunofluorescence for Prdm1 (**A**, Ab from Cell Signaling Technology) and the myoblast marker Pax7 (**B**). The myoblasts illustrated here all co-expressed Prdm1 and Pax7. **C**. Quantitative analysis of proliferating E4 cultures prior to myotube formation. Cultures were double immunostained for Prdm1 (Ab from Cell Signaling Technology) and Pax7 and staining patterns were quantified. Cells positive for both Pax7 and Prdm1 were the most abundant cell type in somitic (S), forelimb bud (F), and hindlimb bud (H) cultures (yellow bars). A small percentage of cells was positive for Pax7 but negative for Prdm1 (red bars). Some cells were also negative for Pax7 but positive for Prdm1 (green bars) or negative for both (gray bars). Bar  = 10 µm.

To determine if Prdm1 was expressed in myoblasts, as well as in MyHC-expressing cells, we next examined E4 somite and limb bud cultures by double immunofluorescence for Prdm1 with the Cell Signal Technology antibody and an antibody specific for the myoblast marker Pax7 [32], [33] (Fig. 5). We found that ∼40% of the cells in E4 somite, hindlimb bud, and forelimb bud cultures had nuclei that were immunostained for both Pax7 and Prdm1 (Fig. 5A, B). Furthermore, cells that were Pax7-positive but Prdm1-negative were rare at only 3–6% of the total cells, thus ∼80–90% of the all the cells that were Pax7-positive were also Prdm1-positive. In addition, we found that about a quarter of the cells were Prdm1-positive/Pax7-negative or doubly negative (Fig. 5C). The identity of these cells is not known, but non-myogenic cells such as fibroblasts are certainly in the cultures. Thus, Prdm1 was expressed by a large majority of the Pax7-positive myoblasts, suggesting that Prdm1 was expressed not only in the terminally differentiated, MyHC-expressing cells but at earlier stages of myogenesis as well.

**Figure 6:**
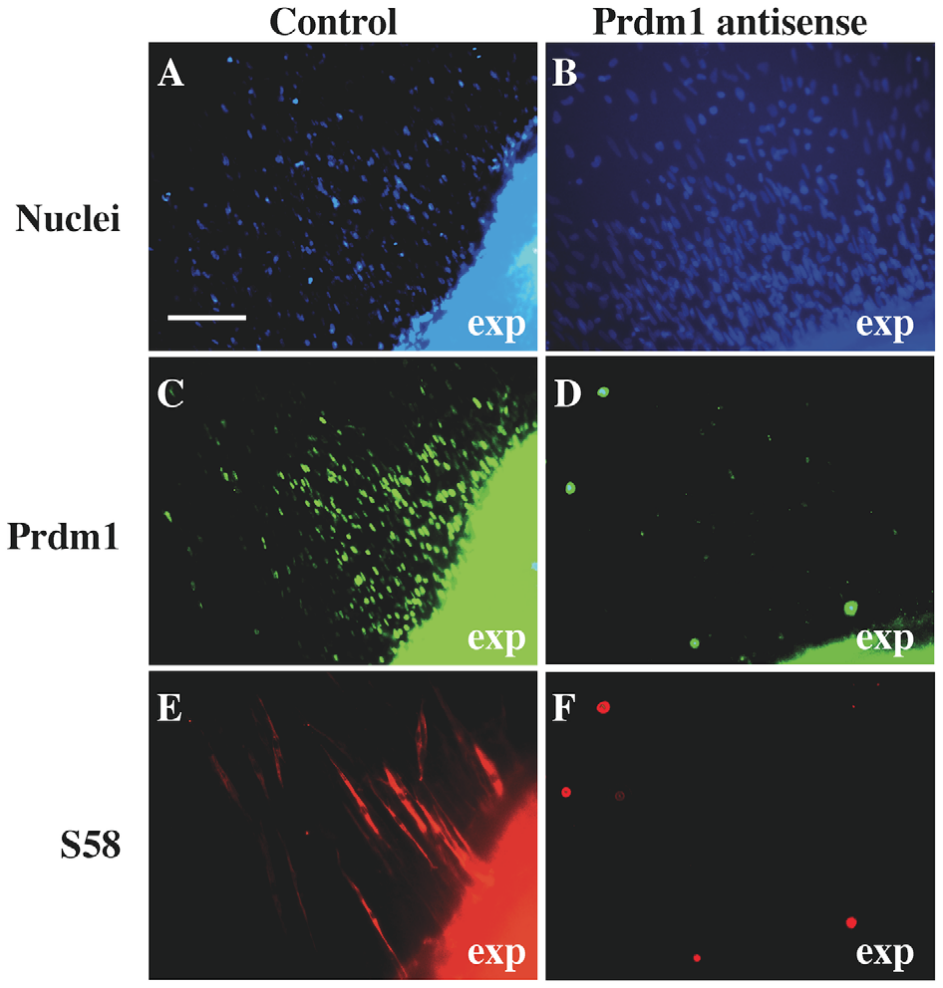
Antisense oligonucleotides decreased Prdm1 immunostaining and inhibited expression of fast and slow MyHCs in somite explant cultures. Somite tissues were dissected from E4 embryos (HH stage 23–25), cultured for 2 days, and analyzed for expression of Prdm1 and MyHC isoforms. **A, C, E**. In the presence of a non-specific oligonucleotide, cells derived from the main explant formed a surrounding monolayer of cells (A, nuclei) and both Prdm1-positive cells (C, Ab from Cell Signaling Technology) and MyHC-expressing myocytes (E, S58-positive) were found among these cells. The main tissue explant (exp) is to the lower right of each panel. **B, D, F**. In the presence of Prdm1 antisense nucleotides, formation of the monolayer was not affected (B, nuclei), but expression of both Prdm1 (C, Ab from Cell Signaling Technology) and slow MyHC(s) (F, mAb S58) was inhibited. Staining with mAb F59, and thus fast MyHC expression, was also inhibited by Prdm1 knockdown (not shown). See text for quantitative analysis. Bar in A = 75 µm.

Finally, to begin to determine the function of Prdm1 in myogenesis, we examined fast and slow MyHC expression after antisense knockdown of Prdm1 expression in E4 somite explant cultures (Fig. 6). During the first 1–2 days after explants were established, we found that cells migrated from the main explant to form a surrounding monolayer of cells and that Prdm1-positive cells and MyHC-expressing myocytes formed among the cells in the monolayer. Treatment of the somite cultures with a control oligonucleotide that was not specific for Prdm1 had no affect on formation of either the surrounding monolayer of cells or on the differentiation of fast/slow myocytes (Figs. 6A, C, E). This pattern of differentiation in which cells in the surrounding monolayers were almost all Prdm1-positive and a fraction of the cells were MyHC-expressing myocytes was seen consistently in nine independent explants treated with the control oligonucleotide. We also examined ten independent somite explant cultures that had been treated with Prdm1 antisense oligonucleotides (see Methods) for 1 or 2 days. In seven of these explants, we found that formation of the surrounding monolayer of cells appeared to be unaffected (Fig. 6B), whereas staining was largely eliminated for Prdm1 (Fig. 6D), mAb S58 (Fig. 6F), and mAb F59 (not shown). Using Fisher's exact test, the difference between the consistent appearance of Prdm1 and MyHC in 9 of 9 control cultures was significantly different from the opposite findings in 7 of the 10 antisense cultures (P<0.01). Though we cannot explain why the antisense knockdown protocol sometimes failed, the overall findings support the conclusion that knockdown of Prdm1 expression was accompanied by knockdown of expression of F59-reactive fast MyHC(s) and S58-reactive slow MyHCs 2 and/or 3.

## Discussion

In cultures of embryonic E4 somite, embryonic E4 limb, and fetal E12 limb cells, we found that Prdm1 was expressed in a large majority of Pax7-positive myoblasts and in all differentiated MyHC-expressing cells, irrespective of the developmental stage of cell donor or the pattern of fast and slow myosin heavy chains expressed in the differentiated cells that were formed. Thus, Prdm1 was expressed in myogenic cells prior to terminal differentiation and expression was not limited to cells that expressed slow myosin heavy chain. In addition, the antisense experiments provided evidence that Prdm1 was required for differentiation of somitic myocytes in culture. Somitic myocytes are the earliest myocytes to form in the avian embryo. Below, we discuss our results in relation to current understanding of the multiple stages of chicken myogenesis and to previous studies, largely in zebrafish, of Prdm1 function in myogenesis.

In the developing chicken, multiple types of somitic, embryonic, and fetal myoblasts arise and form different fast and slow types of myofibers [16], [17], [24]–[27]. The first chicken skeletal muscle cells form in the somitic myotome. Mature myotomal fibers in the chicken embryo uniformly co-express the embryonic fast MyHC along with all three of the slow MyHCs, whereas the neonatal and adult fast MyHCs are not expressed in myotomal fibers [21]. The adaxial somites of zebrafish also initially co-express fast and slow MyHCs [34]. Of the MyHC isoforms expressed in chick myotomal fibers, only slow MyHC2 requires functional innervation for expression [21].

In our studies of chick embyro somite explants (which contained neural tube), we found that all myocytes reacted with both mAb F59 (which reacts with embryonic fast MyHC, as well as all other chicken fast MyHCs) and mAb S58 (which reacts strongly with slow MyHCs 2 and 3 and weakly with slow MyHC1). This staining pattern is consistent with the pattern of fast and slow MyHC co-expression found *in vivo* [21]. In addition, we found that all somite-derived myocytes expressed Prdm1 protein *in vitro*. In contrast to a previous whole mount *in situ* hybridization study that did not detect Prdm1 mRNA expression in chick somites [35], which may have been due to lack of probe penetration, we found that Prdm1 mRNA was expressed in somite tissues and that Prdm1 protein was expressed in the somitic myotome *in vivo*. Furthermore, we found that expression of both fast and slow MyHC isoforms was inhibited by antisense knockdown of Prdm1 in somite cultures, suggesting that Prdm1 was required for MyHC expression in somite-derived muscle cells.

Because many of the conclusions of this work rest on the specificity of the antibodies for chicken Prdm1, we have presented multiple lines of evidence that support the specificity of the Prdm1 staining, including: (i) antibodies that were made to two different epitopes that are conserved in chicken Prdm1 showed similar staining in chicken cells; (ii) in contrast, an antibody that was made against a human Prdm1 epitope that is not conserved in chicken Prdm1 failed to stain chicken cells; (iii) on immunoblots, the antibodies recognized a single band of ∼100 kDa, which is the predicted size of chicken Prdm1 (Fig. 1); (iv) Prdm1 mRNA was detectable by RT-PCR (using primers that spanned exon junctions) in all cell cultures in which Prdm1 immunostaining was found and in all tissue regions from which cells were obtained for culture; (v) incubation with the C-terminal peptide used for immunization blocked immunostaining by the corresponding antibody (not shown, see Materials and Methods); (vi) on immunoblots and in cultures, staining was eliminated in controls that either lacked the Prdm1 antibody (no primary antibody), had non-immune serum instead of the Prdm1 antibody, or had a non-Prdm1 antibody; and (vii) Prdm1 immunostaining was almost completely eliminated by treating somite-containing explants with antisense RNAi oligonucleotides that were designed to specifically target Prdm1 mRNA, whereas staining was not affected by treatment with a scrambled RNAi that was not specific to Prdm1.

In addition to our results with somitic myocytes, we found that Prdm1 was also expressed in myoblasts obtained from embryonic (E4) limbs, as well as in the differentiated fast and fast/slow myocytes and myotubes that were formed from these embryonic myoblasts. Two main types of embryonic myoblasts are obtained from E4 limbs: 60–70% of the myoblasts are committed to form small (usually 1–3 nuclei) myocytes or myotubes that express only fast MyHC(s) and the remaining 30–40% of the myoblasts are committed to form myotubes that co-express both fast and slow MyHCs [24], [25], [30]. We confirmed the formation of these fast (F59-positive, S58-negative) and fast/slow (F59-positive, S58-positive) types of myotubes in E4 cultures, and we found that Prdm1 was expressed in the nuclei of both types of myotubes. Because none of the three slow MyHC isoforms is expressed by fast myotubes in E4 cultures [31], this result showed that Prdm1 expression was not limited to differentiated cells that expressed slow MyHC, but was also found in myotubes that expressed only fast MyHC(s). In addition, we found that Prdm1 was expressed in 80–90% of the Pax7-positive myoblasts in the E4 cultures. This result suggests that Prdm1 was expressed by both types of myoblasts in E4 cultures, *i. e.*, the ∼60–70% [24], [25] of the total myoblasts which were of the fast type and the remainder which were of the fast/slow type.

Finally, we found that Prdm1 was also expressed in fetal E12 myoblasts and the myotubes they formed in culture. Fetal myoblasts are distinct from and replace embryonic myoblasts as development proceeds [16], [17]. Fetal myoblasts form large multinucleate myotubes that initially express only fast MyHC(s), though, after 7–10 days of differentiation, a percentage of the fetal myotubes begin to co-express slow MyHC1 (but not slow MyHC2 or 3) along with the fast MyHC(s) [31]. We examined fetal cultures within 4–5 days of differentiation at which time only fast MyHCs were expressed in the myotubes. Though it was unexpected to find Prdm1 expression in the fetal myoblasts and myotubes, our combination of mRNA, immunoblotting, and immunocytochemical studies (with multiple antibodies to different epitopes) provided confidence that Prdm1 was expressed in the fetal limb-derived myogenic cells. In fetal cultures, as in embryonic cultures, therefore, Prdm1 expression was not limited to cells that expressed slow MyHC(s), but was also found in myotubes that expressed only fast MyHC(s).

Our results suggest that the developmental pattern of Prdm1 expression differs between chickens and zebrafish. In the somites of zebrafish, for example, Prdm1 expression is limited to the adaxial somite cells (which later migrate to form the superficial layer of slow myofibers) and is not found in the fast myofibers [1], [7], [9]. Lamprey somites have a similar adaxial pattern of Prdm1 expression [4]. In the chick embryo, in contrast, we found that Prdm1 was expressed both throughout the mature somitic myotome (Fig. 2D) and in all somitic myocytes that formed in culture. In addition, expression of zebrafish Prdm1 is limited to a subset of all of the myofibers that express slow MyHC(s), whereas we found that chicken Prdm1, at least in cultures, was expressed in all myotubes, including those that did not express slow MyHC(s) such as the embryonic and fetal fast myotubes. In the mouse, the role of Prdm1 in myofiber formation and diversification has not been analyzed in detail [4]–[6], [36]. Similar to what we found in the embryonic chicken, Prdm1 is expressed throughout the somitic myotomes of the mouse [36], [37]. In somites of E10.5 Prdm1-null mouse embryos, the domain of *Fgf8* gene expression is expanded, but the possible effects of Prdm1 inactivation on myotomal structure and MyHC expression in mouse somites have not been reported [36]. Additional studies have shown that, in the mouse, Prdm1 is required for formation of posterior limb structures including musculature [4], though the effect of Prdm1 inactivation on limb myofiber diversification was also not specifically examined.

Though our study clearly points to a role for Prdm1 in the different stages of chicken myogenesis, our study was limited and much remains to be determined before a complete comparison across evolution can be contemplated. For example, it remains to be determined if myogenesis in E4 and E12 limb cultures or *in ovo* is affected by Prdm1 knockdown, but these experiments were beyond our resources at this time. Also, the expression pattern of Prdm1 in developing chicken limbs needs to be studied throughout development to determine if Prdm1 becomes limited to posterior limb bud regions as found in the mouse embryo and suggested by the previous *in situ* hybridization study in chicken embryos [35]. Experiments are also needed to determine in which of the somitic, embryonic, and fetal myoblasts and myotubes or regions within the developing somites and limbs that Prdm1 expression is dependent on Hedgehog family signaling.

In summary, we provide evidence that in chicken myogenic cell cultures, Prdm1 was expressed in most Pax7-positive myoblasts and in all differentiated muscle cells, irrespective of the developmental stage of cell donor or the pattern of fast and slow myosin heavy chains expressed in the differentiated cells that were formed. Thus, Prdm1 was expressed in chicken myogenic cells prior to terminal differentiation and, after differentiation, Prdm1 expression was not limited to cells that expressed slow myosin heavy chain isoforms. In addition, Prdm1 appeared to be required for differentiation of the somitic myocytes, which are the earliest myocytes to form in the avian embryo. In contrast to zebrafish, where expression of Prdm1 is limited to a subset of slow MyHC-expressing cells that originate from adaxial somite cells, Prdm1 expression in the chicken embryo and cultured myogenic cells was much more widespread. Thus, Prdm1 is likely required at more stages of myogenesis in avian than in fish embryos.

## Methods

### Cell nomenclature

We used our previous nomenclature for myogenic cells in the developing chicken [31]. Myoblasts are myogenic, mononucleate, and mitotic cells that do not express skeletal muscle MyHC(s). Myotubes are non-mitotic, do express skeletal muscle MyHC(s), and can be either mononucleate (also known as myocytes) or multinucleate. The embryonic period of development is a period of morphogenesis that, for the chicken, lasts until about E8 -10, and the fetal period of development is a period of growth that follows until hatching. Embryonic myoblasts, isolated from the E4–5 developing bird, and fetal myoblasts, isolated from the E12 developing bird, correspond to the early and late muscle colony-forming myoblasts defined by Hauschka and coworkers [16], [17].

### Antibodies

Previous studies have described both mAb F59, a mouse IgG1 that reacts with embryonic, neonatal, and all adult fast MyHC isoforms of the chicken, and mAb S58, a mouse IgA that reacts strongly with slow MyHC2 and slow MyHC3 and weakly with slow MyHC1 [21], [22], [29], [30], [38]. Mouse anti–glyceraldehyde 3-phosphate dehydrogenase (GAPDH) was from Research Diagnostics (Flanders, NJ) or Fitzgerald Industries (Concord, MA) and monoclonal mouse anti-Pax7 was from the Developmental Studies Hybridoma Bank (Iowa City, IA). To detect Prdm1 by immunoblotting and immunocytochemistry, we used a monoclonal rabbit-anti-Prdm1 (#9115, Cell Signaling Technology, Danvers MA, USA) that was prepared against a human epitope centered on Val90 [Swiss-Prot: O75626.1]; the corresponding epitope region of chicken Prdm1 [ENSGALT00000024824] differs by only one amino acid, whereas that for the mouse Prdm1 [AAI29802.1] has two differences from the human in this region. For immunocytochemistry, we also used two antibodies prepared against the Prdm1 C-terminal amino acid sequence (KVKQETVEPMDP) that is identical in human, mouse, and chicken Prdm1. These two additional antibodies were a polyclonal goat-anti-Prdm1 (ab13700, Abcam, Cambridge MA, USA) and a polyclonal rabbit-anti-Prdm1 (custom preparation by GenScript, Piscataway NJ, USA). The nuclear immunostaining found with these antibodies was blocked by incubation with the immunizing peptide and no staining was found when any of primary antibodies was omitted or replaced with non-immune serum (not shown). We also tested an additional polyclonal rabbit-anti-Prdm1 (NB100-56264, Novus Biologicals, Littleton CO, USA). However, this antibody was prepared against a 15 amino acid human epitope TQTQSSLKQPSTEK [Genbank: CAI18902.1] that shared only six identical amino acids with the corresponding region of the chicken Prdm1 (TQTHVNPKQHSADKD), and we found that this antibody did not react with chicken Prdm1.

### Immunoblotting

Immunoblotting was performed much as described previously (29, 31). From E4 embryos (HH stage 23–25), the forelimb buds, hindlimb buds, and trunk tissue from between the fore- and hindlimbs were dissected and homogenized on ice in a buffer (recommended by Cell Signaling Technology) consisting of 20 mM Tris pH 7.5, 150 mM NaCl, 1 mM EDTA, 1 mM EGTA, 1% Triton X-100, 2.5 mM Sodium Pyrophosphate, 1 mM β-glycerophosphate, Sodium Vanadate, and Calbiochem III Protease Inhibitors. From E12 embryos, the thigh and upper wing were dissected, skinned, and similarly homogenized. Homogenized samples were subjected to SDS-PAGE in 12% gels, transferred to nitrocellulose, and incubated with primary antibodies for Prdm1 or GAPDH at 1.25 µg/ml overnight. Primary antibody binding in Fig. 1 was detected with Alexa-680-conjugated secondary antibodies with appropriate species specificity (Invitrogen goat-anti-rabbit #A21076 and goat-anti-mouse #A21057) [39].

### Cell culture

For somite explant cultures, thoracic tissue from E4 embryos was excised from between the fore- and hindlimbs in Hank's balanced salt solution. The resulting somite region tissue was cut in half along the length of the neural tube and then crosswise into small segments containing one or more somites. The explants were cultured in equal parts fresh and conditioned [25] medium consisting of F-10 (Invitrogen) supplemented with 5% chick embryo extract, 15% horse serum, glutamine, and 1.2 mM CaCl_2_. Cells from E4 embryonic and E12 fetal fore- and hindlimbs were prepared and cultured as described [30], [31]. Cells of the mouse C_2_C_12_ myogenic cell line were grown as described [39], [40].

### Immunochemical staining

Cultures were fixed in 100% ice-cold methanol for ten minutes, washed with PBS, blocked for 1 h, and incubated with primary antibody (or antibodies) overnight at 4°C. Frozen transverse sections of forelimb bud level somites of E4 embryos (HH stage) were post-fixed with 4% paraformaldehyde. Cultures or sections were incubated with anti-Prdm1 antibody at a dilution of 1∶250 (Cell Signaling) or 1∶100 (Abcam) overnight. Hybridoma supernatants were diluted 1∶10 for mAbs F59 and S58 and 1∶1000 for Pax7. Binding of primary antibodies in Figs. 2, 3 (except panels C and G), 4, 5, 6C and 6D was detected with secondary antibodies conjugated with Alexa-350 (blue, Invitrogen goat-anti-mouse #A11045), Alexa-488 (green; Invitrogen goat-anti-rabbit #A11001), or Alexa-594 (red, Invitrogen goat-anti-mouse #A11005). Binding of mAb S58 in Figs. 3C, 3G, 6E, and 6F was detected with biotinylated goat-anti-mouse IgA (Santa Cruz Biotech #SC-3691) and Texas Red-conjugated avidin (Invitrogen #A820).

### Antisense

Three RNAi oligonucleotides were designed to be specific for the chicken Prdm1 mRNA [Genbank: AB278131] and were synthesized (Stealth RNAi, Invitrogen, Carlsbad, CA, USA). The RNAi sequences were GGACGGAGGCTGATTTCGAAGAGAA (start site at nt 38), GATTTCGAAGAGAAGTGCACGTACA (start site at nt 49), and CCTTGCCAAGGAACTTGACTTTCAA (start site at nt 137). A control oligonucleotide and Lipofectamine 2000 were also from Invitrogen (product #13750070). Lipofectamine 2000-mediated transfection was used to introduce RNAi oligonucleotides into somite explant cultures. For each experiment, 24 pmol of each Prdm1 antisense RNAi (72 pmol total) or 72 pmol of the control oligonucleotide was complexed with Lipofectamine in Opti-MEM (Invitrogen) and then incubated with explant cultures on 35 mm culture dishes for 4 h at 37°C. Prdm1 mRNA is expected to have a short half-life [41]. Cultures were fixed and examined by immunocytochemistry 24 h after the end of the transfection.

### RT-PCR

RNA was isolated from cells or tissues, treated with DNase, reverse transcribed, and PCR amplified [42]. For Prdm1, tissues and cells were analyzed with two different PCR primer sets to increase confidence. The first set of primers for Prdm1 mRNA included a forward primer TCATACCAGCACCTAACAGTGCCT and a reverse primer TCTTCAGTGGGTATGGGAGGGTTT that were designed to give a product of 240 bp. The second set included a forward primer AAGAGCTGCTTGTGTGGTATTGCC and a reverse primer TCACACTGTGCTCCTTCTTTGGGA with a product of 171 bp. For the control GAPDH, the forward primer ATGCCATCACAGCCACACAGAAGA and reverse primer ATTCAGCTCAGGGATGACTTTCCC gave a product of 219 bp. PCR amplification used 30 s cycles of denaturation at 95°C, annealing at 57°C, and extension at 72°C. For cDNA products produced with the first set of Prdm1 primers, restriction enzyme analysis with either SgrAI (predicted to cut at nucleotide 159 of the Prdm1 amplification product) or BsteII (nucleotide 82) was used to confirm the identify of the amplified Prdm1 cDNA (not shown).
